# Vaccination as a Remedy for Whooping-Cough

**Published:** 1885-11

**Authors:** 


### VACCINATION AS A REMEDY FOR WHOOPING COUGH.

DR. F. W. ENTRIKEN in the *Lancet and Clinic* says for "over a quarter of a century he has been perfectly familiar with the practice" (vaccinating with vaccine lymph to cure pertussus,) and that he knows a number of regular physicians who practice it, and that moreover, "Prof. Chapman taught it in Philadelphia in 1828." So confident is Dr. Entriken that vaccination will cure the whooping cough that he says he has "often, when no smallpox was near, and no journey was prospective, advised postponing vaccination till the child contracted whooping cough." "I have vacciniated, for this purpose" says the doctor, "several hundred children, and almost always with happy results,—usually, a speedy cure." Unfortunately Dr. E. does not enlighten the profession as to *how* the cure is effected; he only says: "I think it best to give the usual treatment until the febrile stage has passed, then vaccinate, the new disease set up by the virus will then usually arrest the nervous and other distressing symptoms, that are so apt to linger and perpetuate the morbid condition."

A writer in the *Lancet and Clinic*, however, seems to assume that it is an application of "bactero—therapy"—and that the "organisms" of vaccinia, have a fondness for—as an article of diet, those of the whooping cough, and accordingly "go for" them, and devour them,—to the patient's relief.

We give our readers the benefit of the above, as one of the "new remedies" proposed, or, according to Dr. E.—one of the newly revived old remedies—somewhat like some of Park Davis & Co.'s new remedies, Jamaica dog-wood for instance. The curious will find an account of this new remedy in the secondary list of the U. S. Dispensatory for 1850, attributed to "Prof. Hamilton," in 1841. It is a little singular that if the process of *curing* whooping cough by vaccination was known and practiced by Prof. Chapman in 1828, and *especially* if it is, or was then, as sure a thing as Dr. Entriken asserts it is—it has never come into general use. There is no more distressing malady than whooping cough, and none more obstinate in its resistance to *all* treatment at times, than whooping cough, and if vaccination will even mitigate its severity, it will indeed prove a boon to unborn generations, empirical though it be.

